# Supplementary material for: Mucosal application of the broadly neutralizing antibody 10-1074 protects macaques from cell-associated SHIV vaginal exposure
Source: Nat Commun. 2023 Oct 6;14:6224. doi: 10.1038/s41467-023-41966-4 (PMC10558491; doi:10.1038/s41467-023-41966-4)
Supplement: Supplementary file 1 — Supplementary Information [file 41467_2023_41966_MOESM1_ESM.pdf]

Supplementary Information for

Mucosal application of the broadly neutralizing antibody 10-1074 protects macaques from cell-associated SHIV vaginal exposure

Karunasinee Suphaphiphat et al.

The PDF file includes:

Figures S1 to S3

Tables S1 to S7

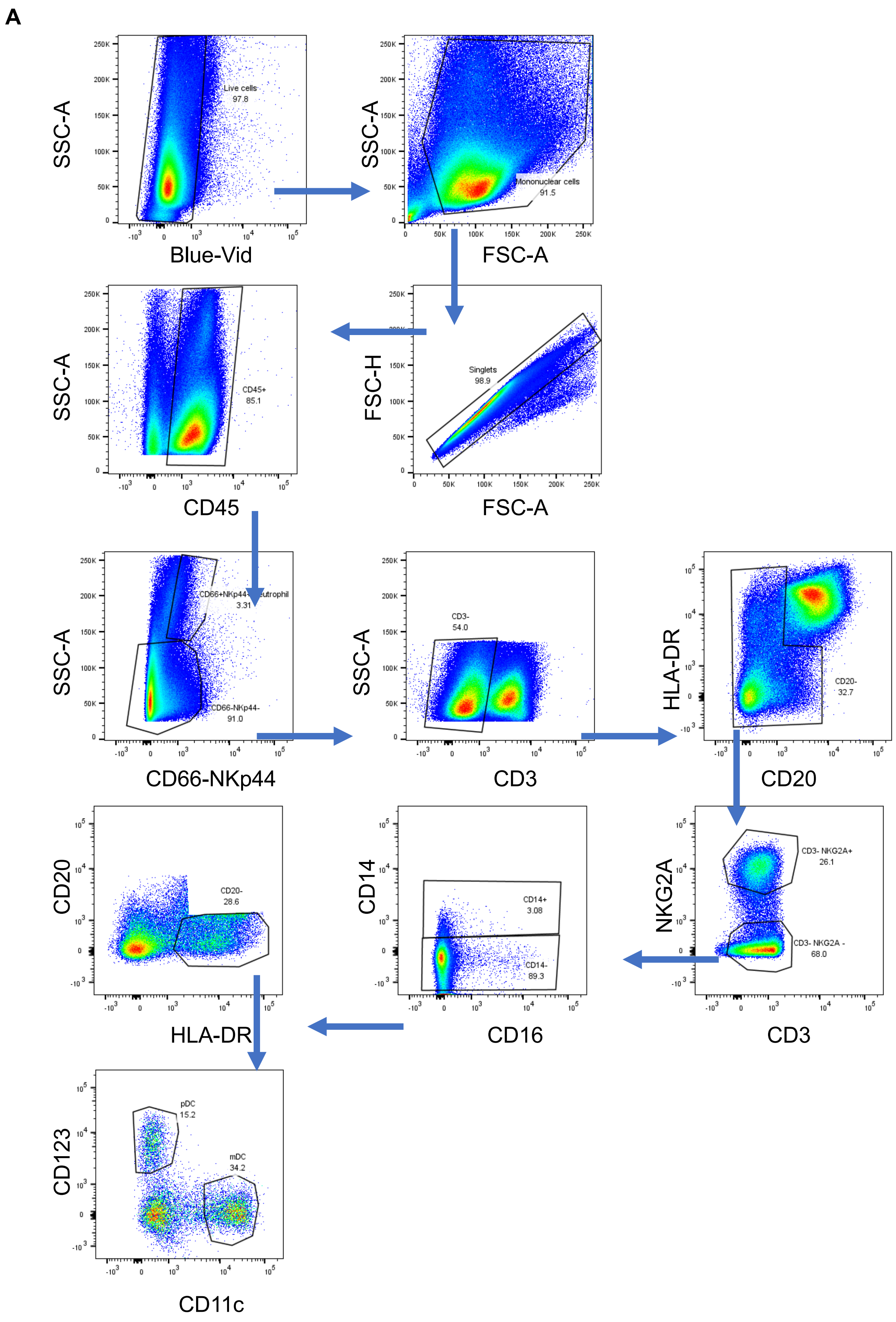
A
SSC-A
Blue-Vid
Live cells
97.8
FSC-A
Mononuclear cells
91.5
FSC-H
Singlets
98.9
CD45
CD45+
85.1
CD66-NKp44
CD66+NKp44- Neutrophil
3.31
CD66-NKp44-
91.0
CD3
CD3-
54.0
HLA-DR
CD20
CD20-
32.7
NKG2A
CD3- NKG2A+
26.1
CD3- NKG2A -
68.0
CD14
CD16
CD14+
3.08
CD14-
89.3
CD20-
28.6
CD123
CD11c
pDC
15.2
mDC
34.2

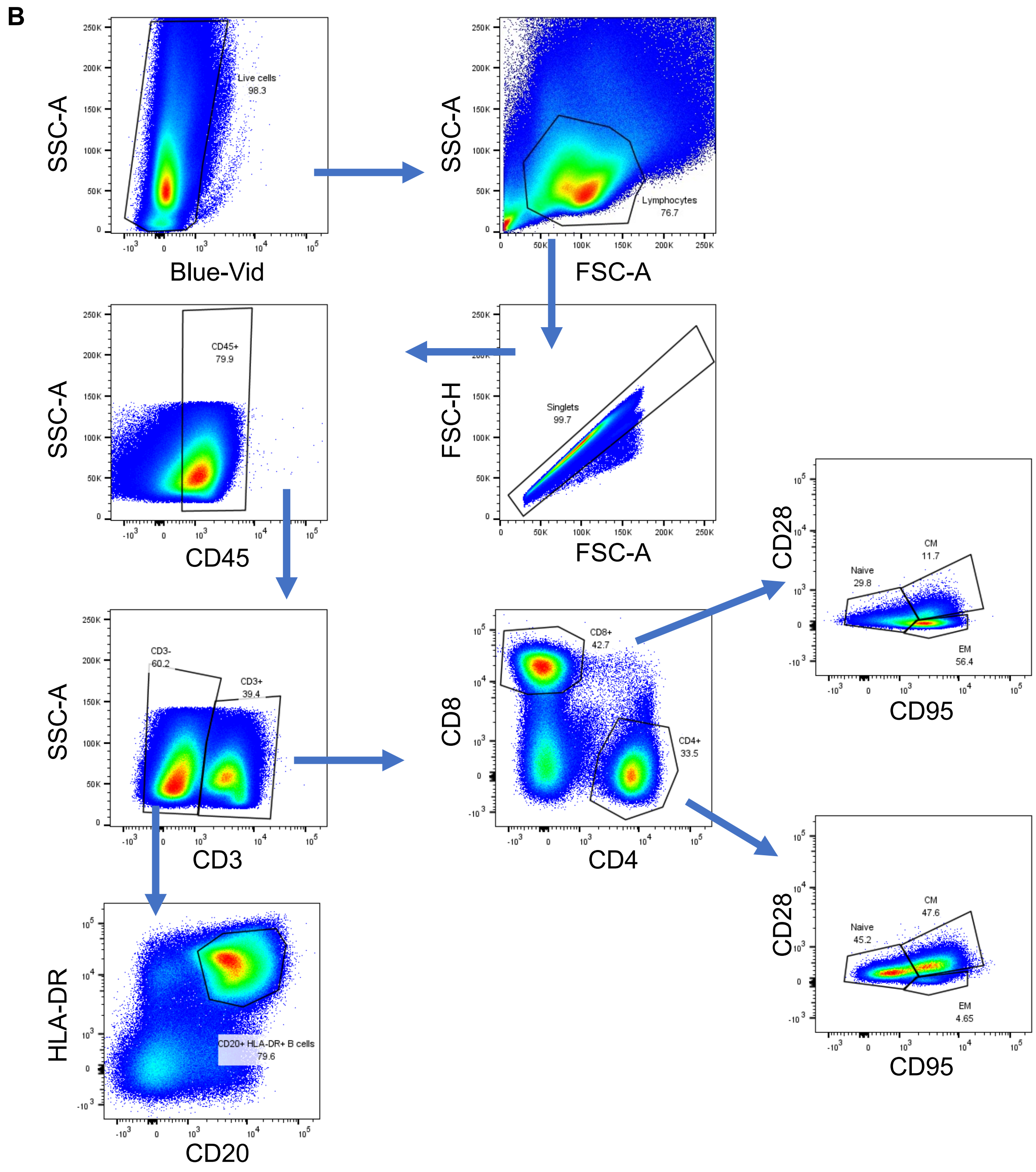

Fig. S1. Gating strategy used to characterize the phenotype of SHIV$_{162P3}$ infected cells. Representative gating strategy used to characterize (**A**) myeloid and (**B**) lymphocyte populations. After gating on live, single cells, and CD45^{+} cells, we identified: (**A**) neutrophils (CD66^{+}NKp44^{+}); monocytes/macrophages (CD66^{-}NKp44^{-}CD3^{-}CD20^{-}NKG2A^{-}HLA-DR^{+}CD14^{+}); plasmacytoid dendritic cells (CD66^{-}NKp44^{-}CD3^{-}CD20^{-}NKG2A^{-} HLA-DR CD14^{-}CD123^{+}) and myeloid dendritic cells CD66^{-}NKp44^{-}CD3^{-}CD20^{-}NKG2A^{-} HLA-DR CD14^{-}CD123^{+}). (**B**) B lymphocytes (CD3^{-}HLADR^{+}CD20^{+}) and T lymphocytes (CD3^{+}). Both CD4^{+} T cells (CD3^{+}CD8^{-}CD4^{+}) and CD8^{+} T cells (CD3^{+}CD8^{+}CD4^{-}) were further classified as

naïve ($CD28^+CD95^-$), central memory ($CD28^+CD95^+$), and effector memory ($CD28^-CD95^+$) cells.

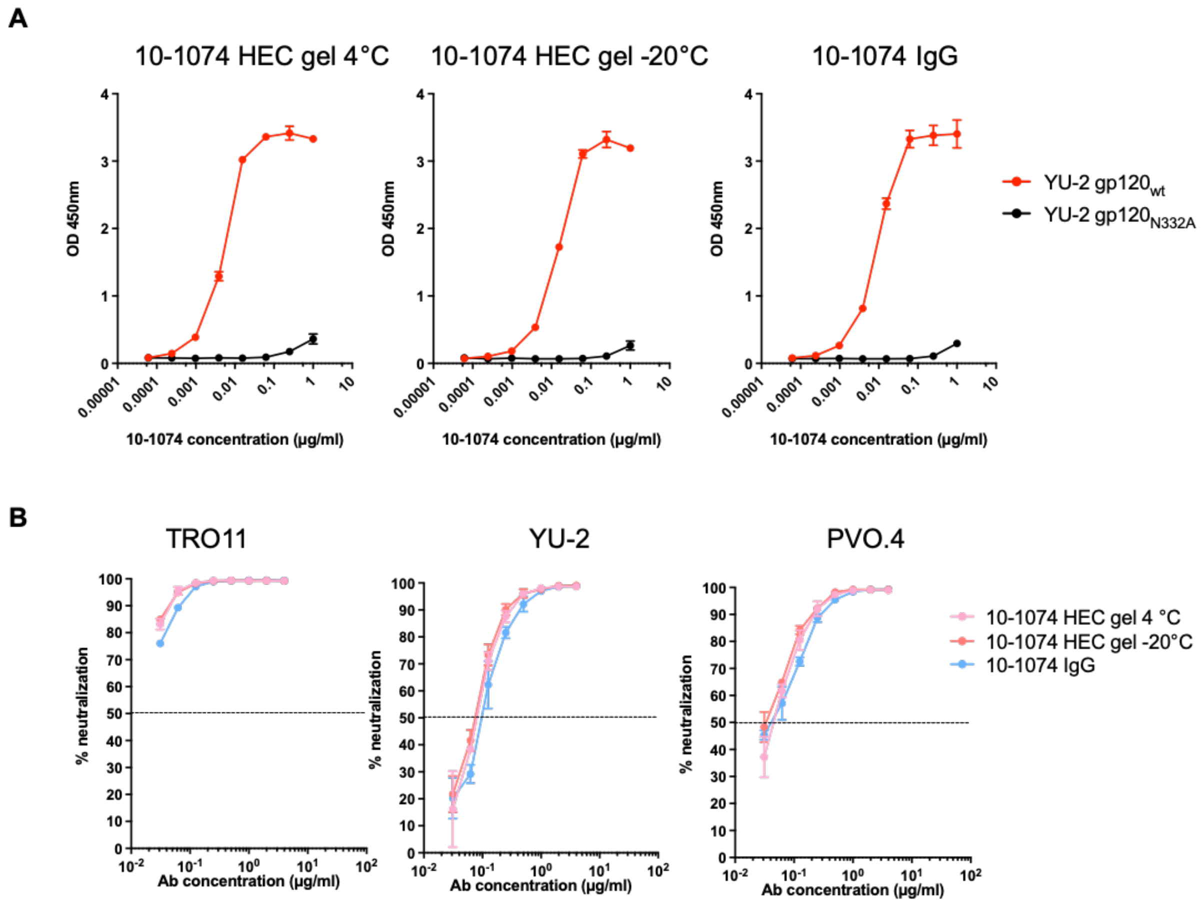

C

	TRO11	PVO.4	YU-2
	IC50 (μg/ml)		
**10-1074 IgG**	0.008	0.124	0.470
**10-1074 HEC gel (4°C)**	0.006	0.113	0.308
**10-1074 HEC gel (-20°C)**	0.003	0.062	0.176

Fig. S2. Storage temperature does not affect the 10-1074 HEC gel binding and inhibitory potency.

The 10-1074 Ab was formulated as an HEC gel at a 5 mg/g concentration and stored for 8 weeks at either 4°C or -20°C. (**A**) An enzyme-linked immunosorbent assay (ELISA) was used to detect the binding of the 10-1074 HEC gel to the YU-2 $gp120_{wt}$ and $gp120_{N332A}$ proteins. The monoclonal IgG 10-1074 Ab stored at 4°C was used as a positive control. The mean and standard deviation (SD) of n = 1 representative experiment performed in triplicate are shown. (**B**) The neutralization activity of three HIV-1 viral strains to 10-1074 IgG and 10-1074 HEC gel was determined in a TZM-bl assay system. The mean and SD of n = 1 representative experiment performed in triplicate are shown. (**C**) The calculated 50% of inhibitory

concentration (IC50) is shown. The assays were performed in triplicate and repeated twice. Source data are provided as a Source Data file.

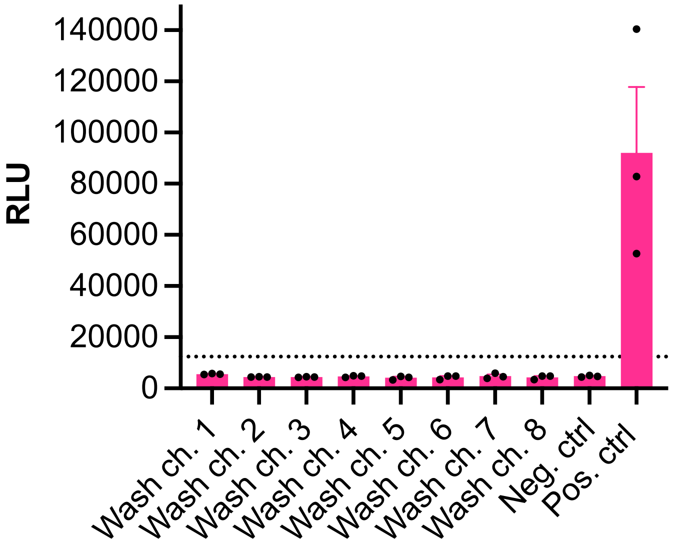

Fig. S3. The cellular inoculum does not contain infectious cell-free viral particles. The supernatant of the final wash of splenocytes before each of the eight inoculations of cells to macaques was collected and incubated for 48h with 10^4 TZM-bl cells. Infection was recorded as Relative Light Unit (RLU). None of the supernatants transmitted infection to the TZM-bl cells. Negative and positive controls were TZM-bl cells incubated with either DMEM medium or cell-free $SHIV_{162P3}$ virus. The dashed line represents the cut-off of infection. Each bar represents the mean and standard error of the mean (SEM) of n = one representative experiment performed in triplicate. Source data are provided as a Source Data file.

Table S1. The proportion of leukocyte populations among splenocytes from macaque SD01, SD02, and SD03.

	SD01	SD02	SD03
**Total T lymphocytes** (% of CD45+ cells)	65.7%	39.4%	46.8%
**CD4+ T cells** (% of CD45+ cells)	24.5%	13.2%	19.5%
- **Central memory**	24.9%	47.6%	49.9%
- **Effector memory**	0.8%	4.65%	5.96%
- **Naive**	73.8%	45.2%	42.5%
**CD8+ T cells** (% of CD45+ cells)	22%	16.8%	16.8%
- **Central memory**	16%	11.7%	25.2%
- **Effector memory**	15.3%	56.4%	32.4%
- **Naive**	66.8%	29.8%	37.5%
**B cells** (% of CD45+ cells)	22.7%	47.8%	38.2%
**Monocytes** (% of CD45+ cells)	0.16%	0.34%	0.13%
**mDC (CD11c+/CD123-)** (% of CD45+ cells)	0.13%	0.94%	0.16%
**pDC (CD11c-/CD123+)** (% of CD45+ cells)	0.17%	0.43%	0.25%
**Neutrophils** (% of CD45+ cells)	1.17%	3.31%	5.87%

Table S2. Neutralization of $SHIV_{162P3}$ by cervicovaginal fluids.

Animal ID	IC50 in TZM-bl cells						
	**1h**	**2h**	**4h**	**6h**	**24h**	**48h**	**72h**
**PK01**	87609	10717	17687	7985	2010	127	49
**PK02**	29795	22614	8942	7029	626	124	76

IC50 values are the CVFs dilution at which relative luminescence units (RLUs) were reduced by 50% compared with virus control wells (no antibody).

Table S3. Time to infection after repeated cell-associated $SHIV_{162P3}$ challenge.

Animal ID	Group	Challenge number at which the animal is considered infected	Week of first positive viremia (post first challenge)	Total number of challenges received
**CM01**	**Placebo gel**	None	-	8
**CM02**	**Placebo gel**	Fourth	5	5
**CM03**	**Placebo gel**	Second	3	3
**CM04**	**Placebo gel**	Seventh	8	8
**CM05**	**Placebo gel**	Fifth	6	7
**CM06**	**Placebo gel**	Fifth	6	7
**CM07**	**10- 1074 gel**	Fifth	6	6
**CM08**	**10- 1074 gel**	None	-	8
**CM09**	**10- 1074 gel**	None	-	8
**CM10**	**10- 1074 gel**	None	-	8
**CM11**	**10- 1074 gel**	None	-	8
**CM12**	**10- 1074 gel**	None	-	8

Table S4. Blood lymphocytes count (10^3/µl) during the protection study follow-up.

Time post 1st challenge (week)	Placebo gel group						10-1074 gel group					
	CM01	CM02	CM03	CM04	CM05	CM06	CM07	CM08	CM09	CM10	CM11	CM12
0	4.4	3.6	3.1	5.3	3.3	3.1	3.5	3.4	3.9	3.7	3.5	2.9
1	3.2	2.8	3.1	4.6	2.9	2.6	2.0	2.2	3.1	3.1	2.5	2.3
2	3.8	3.7	1.8	5.8	2.3	2.6	2.6	2.6	4.9	3.9	4.1	3.5
3	3.9	3.4	2.7	5.2	3.7	3.1	2.6	2.8	5.1	3.7	3.1	3.0
4	2.5	1.4	3.8	4.9	3.2	1.6	2.8	2.1	3.9	3.9	3.1	2.6
5	3.2	2.3	2.4	5.0	n.a.	1.2	1.8	2.5	3.6	3.3	2.3	2.6
6	2.5	4.1	n.a.	4.8	3.3	1.4	3.4	2.4	3.6	3.7	2.6	3.3
7	3.2	3.5	3.2	1.5	5.1	4.7	3.6	2.2	3.3	4.1	2.6	3.3
8	4.1	3.6	2.5	4.0	3.6	3.7	4.1	2.9	4.4	4.5	3.3	3.0
9	4.3	3.7	2.9	6.1	3.3	2.6	3.8	2.9	4.8	4.0	3.8	3.1
10	4.8	3.9	3.1	6.1	2.9	2.4	3.0	2.8	4.4	4.5	2.8	3.5
12	4.4	3.9	3.1	6.2	3.0	3.5	2.9	3.1	4.2	4.4	3.3	2.6
15	3.5	3.3	3.2	5.4	4.6	3.5	3.5	3.2	4.0	4.4	2.9	2.4
19	3.6	3.6	2.8	5.2	4.6	4.8	4.1	3.2	4.1	4.7	3.4	2.6
23	3.6	2.9	2.4	4.5	4.0	4.7	2.8	2.7	5.0	4.8	2.9	2.4
26	2.4	2.8	2.8	3.9	3.8	3.7	2.6	2.6	4.6	4.1	3.2	2.3
31	4.1	3.3	2.8	4.1	4.4	4.0	3.4	3.0	4.8	4.1	4.3	2.8
39	2.2	1.7	2.3	1.3	3.3	3.9	3.2	2.7	3.6	3.5	3.9	2.3
43	3.8	3.4	3.4	2.2	4.3	4.0	3.9	3.2	4.7	4.3	3.5	3.0
52	2.2	2.5	2.5	0.8	3.3	3.6	3.8	2.8	3.8	4.1	3.5	2.0
58	2.8	2.1	2.5	0.2	3.7	2.9	3.8	2.7	3.5	1.6	1.8	1.6

n.a. = not available

Table S5. Cynomolgus macaques characteristics.

Group	ID	Sex	MHC class 1	MHC class 2	Age at beginning of study (year)	Weight at inclusion (kg)
Splenocyte donors	SD01	male	H3-H1	H7	3.2	2.48
	SD02	male	H3-H5	H7	3.6	3.67
	SD03	male	H3	H1	6.8	8.6
Placebo gel	CM01	female	H2-H3	H5	2.9	2.78
	CM02	female	H6-H2	H2	2.9	2.91
	CM03	female	H4	H6	2.4	2.2
	CM04	female	H2	H4-H7	2.4	2.48
	CM05	female	H3-H7	H4-H7	2.9	2.62
	CM06	female	H4-H7	H2	2.8	2.67
10-1074 gel	CM07	female	H6	H7	2.9	2.75
	CM08	female	H4-H7	H2	2.9	2.9
	CM09	female	H4-H7	H4-H7	2.9	2.96
	CM10	female	H5	H6	2.8	2.88
	CM11	female	H1-H2	H2	2.4	2.09
	CM12	female	H2	H4-H7	2.4	2.86
PK study	PK01	female	H2-H4-H6	H4-H6	5.6	3.7
	PK02	female	H4-H1	H1	5.4	4.05

Table S6. In vitro virus production by infected splenocytes.

Splenocyte donor	CVF donor	Time point of culture supernatant collection				
		0h	1h	2h	6h	24h
		Viral load (RNA copies/ml)				
SD03+SD01	-	<LOD	<LOD	<LOD	<LOD	<LOD
SD03+SD02	-	<LOD	<LOD	<LOD	<LOD	<LOD
SD03+SD01	CM07	<LOD	<LOD	<LOD	<LOD	<LOD
SD03+SD02	CM07	<LOD	<LOD	<LOD	<LOD	<LOD
SD03+SD01	CM01	<LOD	<LOD	<LOD	<LOD	<LOD
SD03+SD02	CM01	<LOD	<LOD	<LOD	<LOD	<LOD
SD03+SD01	CM08	<LOD	<LOD	<LOD	<LOD	<LOD
SD03+SD02	CM08	<LOD	<LOD	<LOD	<LOD	<LOD
SD03+SD01	CM03	<LOD	<LOD	<LOD	<LOD	<LOD
SD03+SD02	CM03	<LOD	<LOD	<LOD	<LOD	<LOD

Thawed splenocytes were washed twice, then cells from two different donors were mixed at a 1:1 ratio, and seeded in a 24 well-plate at the concentration of 10^6 total cells/ml of medium (RPMI + 10% FCS % 1% Pen/Strep solution). Cells were cultured in the absence and in the presence of cervicovaginal fluid (CVF) from four different macaques, two belonging to the control group (CM01 and CM03) and two the Ab-treated group (CM07 and CM08). Supernatants were collected immediately or after 1h, 2h, 6h, and 24h of culture at 37°C in a 5% CO2 in a humidified incubator. Viral production was assessed by RT-qPCR. The lower limit of detection (LOD) was estimated to be 333 copies/mL.

Table S7. List of monoclonal antibodies used to characterize the phenotype of infected splenocyte

Panel	Antibody	Clone	Supplier	Catalog number	Ab volume /test (µl)
1 & 2	CD45-PerCP	D058-1283	BD Pharmingen	558411	2.5
1 & 2	CD3-V500	SP34-2	BD Horizon	560770	3
1	CD4-V450	L200	BD Horizon	560811	3
1 & 2	CD8-BV650	RPA-T8	BD Horizon	563821	2
2	CD11c-APC	S-HCL-3	BD Pharmingen	333144	10
2	CD14-AlexaFluor700	M5E2	BD Pharmingen	557923	1
2	CD16- PE-CF594	3G8	BD Horizon	562293	2
1	CD20-PE-CF594	2H7	BD Horizon	562295	0.5
2	CD20-BV711	2H7	BD Horizon	563126	5
1	CD21-BV711	B-Ly4	BD Horizon	563163	5
1	CD27-PE	MT-271	BD Pharmingen	555441	5
1	CD28-FITC	CD28.2	BD Pharmingen	555728	15
1	CD45RA-PC7	L48	BD Pharmingen	337186	1.5
2	CD66-FITC	TET2	Miltenyi	130-093-132	3
1	CD69-AlexaFluor700	FN50	BD Pharmingen	560739	5
2	CD69-V450	FN50	BD Pharmingen	560740	5

1	CD95-APC	DX2	BD Pharmingen	558814	8
2	CD123-PC7	7G3	BD Pharmingen	560826	2.5
1 & 2	HLA-DR-APC-H7	G46-6	BD Pharmingen	561358	5
2	NKp44*	2.29	Miltenyi	130-092-556	10

*Followed by goat-anti-mouse AlexaFluor488 (ThermoFischer Scientific, catalog n° A-11017, volume used 0,2 µl/test).

All antibodies have been validated on human and cynomolgus macaque PBMCs.
