# Supplementary material for: Mucosal application of the broadly neutralizing antibody 10-1074 protects macaques from cell-associated SHIV vaginal exposure
Source: Nat Commun. 2023 Oct 6;14:6224. doi: 10.1038/s41467-023-41966-4 (PMC10558491; doi:10.1038/s41467-023-41966-4)
Supplement: Supplementary file 3 — Reporting Summary [file 41467_2023_41966_MOESM3_ESM.pdf]

nature portfolio

Corresponding author(s): DAPR NCOMMS-23-21686B

Last updated by author(s): Sep 20, 2023

Reporting Summary

Nature Portfolio wishes to improve the reproducibility of the work that we publish. This form provides structure for consistency and transparency in reporting. For further information on Nature Portfolio policies, see our Editorial Policies and the Editorial Policy Checklist.

Statistics

For all statistical analyses, confirm that the following items are present in the figure legend, table legend, main text, or Methods section.

n/a	Confirmed	
☐	☒	The exact sample size (*n*) for each experimental group/condition, given as a discrete number and unit of measurement
☒	☐	A statement on whether measurements were taken from distinct samples or whether the same sample was measured repeatedly
☐	☒	The statistical test(s) used AND whether they are one- or two-sided<br>*Only common tests should be described solely by name; describe more complex techniques in the Methods section.*
☒	☐	A description of all covariates tested
☒	☐	A description of any assumptions or corrections, such as tests of normality and adjustment for multiple comparisons
☐	☒	A full description of the statistical parameters including central tendency (e.g. means) or other basic estimates (e.g. regression coefficient) AND variation (e.g. standard deviation) or associated estimates of uncertainty (e.g. confidence intervals)
☐	☒	For null hypothesis testing, the test statistic (e.g. *F*, *t*, *r*) with confidence intervals, effect sizes, degrees of freedom and *P* value noted<br>*Give P values as exact values whenever suitable.*
☒	☐	For Bayesian analysis, information on the choice of priors and Markov chain Monte Carlo settings
☒	☐	For hierarchical and complex designs, identification of the appropriate level for tests and full reporting of outcomes
☒	☐	Estimates of effect sizes (e.g. Cohen's *d*, Pearson's *r*), indicating how they were calculated

Our web collection on statistics for biologists contains articles on many of the points above.

Software and code

Policy information about availability of computer code

Data collection	No software was used for data collection
Data analysis	Data was analyzed using GraphPad Prism version 9.2.0 software. Flow cytometry data were acquired on an LSRFortessa (BD Biosciences) using BD FACSDiva software (BD Biosciences) and analyzed with Flowjo version 10 (BD Biosciences).

For manuscripts utilizing custom algorithms or software that are central to the research but not yet described in published literature, software must be made available to editors and reviewers. We strongly encourage code deposition in a community repository (e.g. GitHub). See the Nature Portfolio guidelines for submitting code & software for further information.

Data

Policy information about availability of data

All manuscripts must include a data availability statement. This statement should provide the following information, where applicable:

- Accession codes, unique identifiers, or web links for publicly available datasets
- A description of any restrictions on data availability
- For clinical datasets or third party data, please ensure that the statement adheres to our policy

All data are available in the main text or the supplementary materials

Research involving human participants, their data, or biological material

Policy information about studies with human participants or human data. See also policy information about sex, gender (identity/presentation), and sexual orientation and race, ethnicity and racism.

Reporting on sex and gender	n/a
Reporting on race, ethnicity, or other socially relevant groupings	n/a
Population characteristics	n/a
Recruitment	n/a
Ethics oversight	n/a

Note that full information on the approval of the study protocol must also be provided in the manuscript.

Field-specific reporting

Please select the one below that is the best fit for your research. If you are not sure, read the appropriate sections before making your selection.

☒ Life sciences ☐ Behavioural & social sciences ☐ Ecological, evolutionary & environmental sciences

For a reference copy of the document with all sections, see nature.com/documents/nr-reporting-summary-flat.pdf

Life sciences study design

All studies must disclose on these points even when the disclosure is negative.

Sample size	The group size for virus challenge experiments was determined based on previously published results and statistical analysis (Sallé, B. et al. J. Infect. Dis. 2010; Moog, C. et al. Mucosal Immunol. 2014).<br>For in vitro experiments no sample size calculation was performed. In vitro experiments were performed in triplicate and repeated at least twice to allow statistical analysis.
Data exclusions	No data were excluded from the analysis
Replication	In vitro experiments of cell-to-cell transmission and neutralization assay were done in triplicates, and repeated at least twice. All attempts at replication were successful. ELISA assays were done once on duplicate samples. RTqPCR and ELISPOT experiments were performed once and each sample was tested in duplicate. All attempts at replication were successful.
Randomization	Animals were randomly assigned to the two groups. However, they were initially selected to match in terms of age, weigh and MHC.<br>Randomization do not apply to in vitro experiments in this study.
Blinding	For the challenge study blinding was not possible, because the investigators needed to know the specific treatment to apply. The investigators were instead blinded to group allocation during data collection and analysis

Reporting for specific materials, systems and methods

We require information from authors about some types of materials, experimental systems and methods used in many studies. Here, indicate whether each material, system or method listed is relevant to your study. If you are not sure if a list item applies to your research, read the appropriate section before selecting a response.

Materials & experimental systems

n/a	Involved in the study	
☐	☒	Antibodies
☐	☒	Eukaryotic cell lines
☒	☐	Palaeontology and archaeology
☐	☒	Animals and other organisms
☒	☐	Clinical data
☒	☐	Dual use research of concern
☒	☐	Plants

Methods

n/a	Involved in the study	
☒	☐	ChIP-seq
☐	☒	Flow cytometry
☒	☐	MRI-based neuroimaging

Antibodies

Antibodies used

All antibodies are commercially available and described in the Supplementary Informations.

CD45-PerCP, clone D058-1283, Supplier BD Pharmingen, Catalog number 558411, Ab volume/test 2.5 ul
CD3-V500, clone SP34-2, Supplier BD Horizon, Catalog number 560770, Ab volume/test 3 ul
CD4-V450, clone L200, Supplier BD Horizon, Catalog number 560811, Ab volume/test 3 ul
CD8-BV650, clone RPA-T8, Supplier BD Horizon, Catalog number 563821, Ab volume/test 2 ul
CD11c-APC ,clone S-HCL-3, Supplier BD Pharmingen, Catalog number 333144, Ab volume/test 10 ul
CD14- AlexaFluor700, clone M5E2, Supplier BD Pharmingen, Catalog number 557923, Ab volume/test 1 ul
CD16- PE-CF594, clone 3G8, Supplier BD Horizon, Catalog number 562293, Ab volume/test 2 ul
CD20-PE-CF594, clone 2H7, Supplier BD Horizon, Catalog number 562295, Ab volume/test 0.5 ul
CD20-BV711, clone 2H7, Supplier BD Horizon, Catalog number 563126, Ab volume/test 5 ul
CD21-BV711, clone B-Ly4, Supplier BD Horizon, Catalog number 563163, Ab volume/test 5 ul
CD27-PE, clone, MT-271, Supplier BD Pharmingen, Catalog number 555441, Ab volume/test 5 ul
CD28-FITC, clone, CD28.2, Supplier BD Pharmingen, Catalog number 555728, Ab volume/test 15 ul
CD45RA-PC7, clone L48, Supplier BD Pharmingen, Catalog number 337186, Ab volume/test 1.5 ul
CD66-FITC ,clone TET2, Supplier Miltenyi, Catalog number 130-093-132, Ab volume/test 3 ul
CD69-AlexaFluor700, clone FN50, Supplier BD Pharmingen, Catalog number 560739, Ab volume/test 5 ul
CD69-V450 clone FN50, Supplier BD Pharmingen, Catalog number 560740, Ab volume/test 5 ul
CD95-APC ,clone DX2, Supplier BD Pharmingen, Catalog number 558814, Ab volume/test 8 ul
CD123-PC7, clone 7G3, Supplier BD Pharmingen, Catalog number 560826, Ab volume/test 2.5 ul
HLA-DR-APC-H7, clone G46-6, Supplier BD Pharmingen, Catalog number 561358, Ab volume/test 5 ul
NKp44, clone 2.29, Supplier Miltenyi, Catalog number 130-092-556, Ab volume/test 10 ul
Goat-anti-mouse IgG AlexaFluor488, Supplier ThermoFischer Scientific, Catalog number A-11017, Ab volume/test 0,2 ul

Validation

All antibodies were comercially available, specificity had been described by the manufacturer. For human antibodies cross-reactivity with cynomolgus macaques is available from the "Non-Human Primate Reagent Resource" (www.nhpreagents.org). Additionally, antibodies were validated in house using human and macaque PBMCs.

Eukaryotic cell lines

Policy information about cell lines and Sex and Gender in Research

Cell line source(s)

TZM-bl cells were obtained from the NIH AIDS Research and Reference Reagent Program (NIH ARRRP)

Authentication

This cell line has not been authenticated in-house

Mycoplasma contamination

Confirmed negative test for mycoplasma contamination

Commonly misidentified lines
(See ICLAC register)

No commonly misidentified lines were used in this study

Animals and other research organisms

Policy information about studies involving animals; ARRIVE guidelines recommended for reporting animal research, and Sex and Gender in Research

Laboratory animals

This study included a total 17 cynomolgus macaques (Macaca Fascicularis). Three males were involved in splenocyte production (SD01, SD02, SD03), two females in the longitudinal PK study (PK01 and PK02), and twelve females in the efficacy study: six (CM01-CM06) in the placebo gel control group, and six (CM07-CM12) in the 10-1074 HEC gel treated group.
Age of the animals at the beginning of the study is the following (in years):
SD01 (3.2)
SD02 (3.6)
SD03 (6.8)
CM01 (2.9)
CM02 (2.9)
CM03 (2.4)
CM04 (2.4)
CM05 (2.9)
CM06 (2.8)
CM07 (2.9)
CM08 (2.9)
CM09 (2.9)
CM10 (2.8)
CM11 (2.4)
CM12 (2.4)
PK01 (5.6)
PK02 (5.4)

Wild animals	No wild animals were used in the study. All animals used were purposed bread and imported from AAALAC certified breeding centers
Reporting on sex	Only female animals were used for PK and efficacy studies, being the animals subjected to vaginal inoculation
Field-collected samples	No data was collected from the field
Ethics oversight	The study was authorized by the “Research, Innovation and Education Ministry” under registration numbers APAFIS#373 2015032511332650 and #32029-2021061709451888.

Note that full information on the approval of the study protocol must also be provided in the manuscript.

Flow Cytometry

Plots

Confirm that:

☒ The axis labels state the marker and fluorochrome used (e.g. CD4-FITC).

☒ The axis scales are clearly visible. Include numbers along axes only for bottom left plot of group (a 'group' is an analysis of identical markers).

☒ All plots are contour plots with outliers or pseudocolor plots.

☒ A numerical value for number of cells or percentage (with statistics) is provided.

Methodology

Sample preparation	Throughly described in the manuscript
Instrument	Acquisition was performed on a BD LSRII flow cytometer
Software	BD FacsDiva software and FlowJo version 10 was used for acquisition and analysis
Cell population abundance	Throughly described in the manuscript
Gating strategy	Throughly described in the manuscript

☒ Tick this box to confirm that a figure exemplifying the gating strategy is provided in the Supplementary Information.
